# The Effect of Intraoperative Forced Air Warmer Use on Postoperative Nausea, Vomiting, and Shivering Among Laparoscopic Surgery Patients: A Randomized Controlled Trial

**DOI:** 10.7759/cureus.112347

**Published:** 2026-07-09

**Authors:** Tashfain Zafar, Mohammad Hamid

**Affiliations:** 1 Anesthesia, Aga Khan University Hospital, Karachi, PAK

**Keywords:** bair hugger, forced-air warming, hypothermia, laparoscopic surgery, postoperative nausea and vomiting, shivering

## Abstract

Background

Unintended perioperative hypothermia is a frequent side effect of general anesthesia and is related to early postoperative symptoms such as shivering and postoperative nausea and vomiting (PONV). Forced air warming (FAW), as performed with the Bair Hugger system, is commonly used during long procedures for the maintenance of normothermia. However, its effectiveness in reducing these postoperative symptoms in patients undergoing laparoscopic surgery remains uncertain.

Methods

This single-blind randomized controlled trial was conducted at Aga Khan University Hospital, Karachi, between January 1, 2023, and April 10, 2024. Ninety-eight American Society of Anesthesiologists (ASA) I and II patients between the ages of 18 and 65 undergoing laparoscopic surgery were included. These patients were randomized by opaque-envelope allocation technique to the FAW group and the control group (non-FAW). Core temperature was monitored with a nasopharyngeal temperature probe at 30-min intervals. Outcome variables like shivering (BSAS) and PONV scores were recorded at 30 min and 6 h post-extubation in the post-anesthesia care unit (PACU).

Results

Intraoperative use of FAW significantly reduces the incidence of shivering in the first 30 minutes (p=0.004) and, later, when compared with the control group. PONV incidence did not differ at 30 min (p=0.232) and at 6 h (p=0.673).

Conclusions

Forced air warmers help in maintaining intraoperative temperatures and significantly reduce the incidence of postoperative shivering in laparoscopic surgeries, but have no significant effect on nausea and vomiting.

## Introduction

Hypothermia is defined as a condition in which the body's central temperature drops to 36 °C or lower. Unintended intraoperative hypothermia is a frequent and preventable anesthetic complication. It occurs in 50-90% of patients undergoing general anaesthesia [1]. Hypothermia and a decrease in core temperature can lead to adverse physiological consequences, including up to a 400% increase in oxygen consumption [2], shivering, impaired coagulation, delayed anesthetic drug metabolism, and a heightened risk of wound infection [3,4]. It may also cause delayed recovery, greater blood loss, and extended hospital stay. These outcomes negatively affect both patient prognosis and healthcare costs [5].

Laparoscopic surgeries, though minimally invasive, present a significant risk of hypothermia due to multiple factors. These include exposure to low ambient temperature in the operating room and insufflation of cold, dry carbon dioxide into the peritoneal cavity. Anaesthetic-induced vasodilation and redistribution hypothermia after induction also play roles [6,7]. Postoperative shivering and postoperative nausea and vomiting (PONV) are among the most distressing complications during recovery, both associated with perioperative hypothermia [8,9].

Forced-air warming (FAW) systems, such as Bair Hugger, are widely used to prevent hypothermia. They deliver convective heat and are endorsed by international perioperative guidelines for maintaining normothermia [10,11]. Multiple studies highlight FAW's benefits in reducing perioperative hypothermia. However, its influence on postoperative shivering and PONV incidence in laparoscopic surgery remains inconsistent.

Prevention of shivering is especially important in patients with ischemic heart disease, as shivering can trigger myocardial ischemia. FAW is rarely used in laparoscopic surgeries due to the short duration and the cost. Some studies report significant reductions in shivering with FAW, while others find little or no effect on PONV [12-14].

In the last five years, several randomized controlled studies have tested different intraoperative warming strategies for preventing inadvertent hypothermia during laparoscopic procedures. Warming methods in these studies include forced-air warming, prewarming with intraoperative warming, electric blankets, warming mattresses, and warmed IV fluids. Many studies showed positive results for maintaining normothermia and preventing shivering in the postoperative period. However, results for preventing nausea and vomiting varied [15,16].

Given the limited research and debates in the literature, we conducted a single-blind randomized controlled trial to investigate the effectiveness of forced-air warming in adult patients undergoing laparoscopic surgery. The primary objective was to determine its impact on postoperative shivering. Secondary objectives were to assess its effect on PONV and intraoperative core body temperature.

## Materials and methods

This study was approved by the Ethical Review Committee (ERC) of Aga Khan University Hospital, Project ID. 2022-7343-22927, and the study was registered on ClinicalTrials.gov on 6 October 2022 (Registration # NCT05426278). A single-blind randomized controlled trial was conducted at our institute from January 1, 2023, to April 10, 2024. Written informed consent was obtained from all participants preoperatively.

Adult patients aged 18-65 years, American Society of Anesthesiologists (ASA) physical status I-II [17], scheduled for elective laparoscopic surgery under general anaesthesia were included, while patients with anticipated difficult airway, BMI >35 kg/m², baseline fever or hypothermia (<36 °C), thyroid disease, medications affecting thermoregulation, pregnancy, or refusal to participate were excluded.

**Figure 1:**
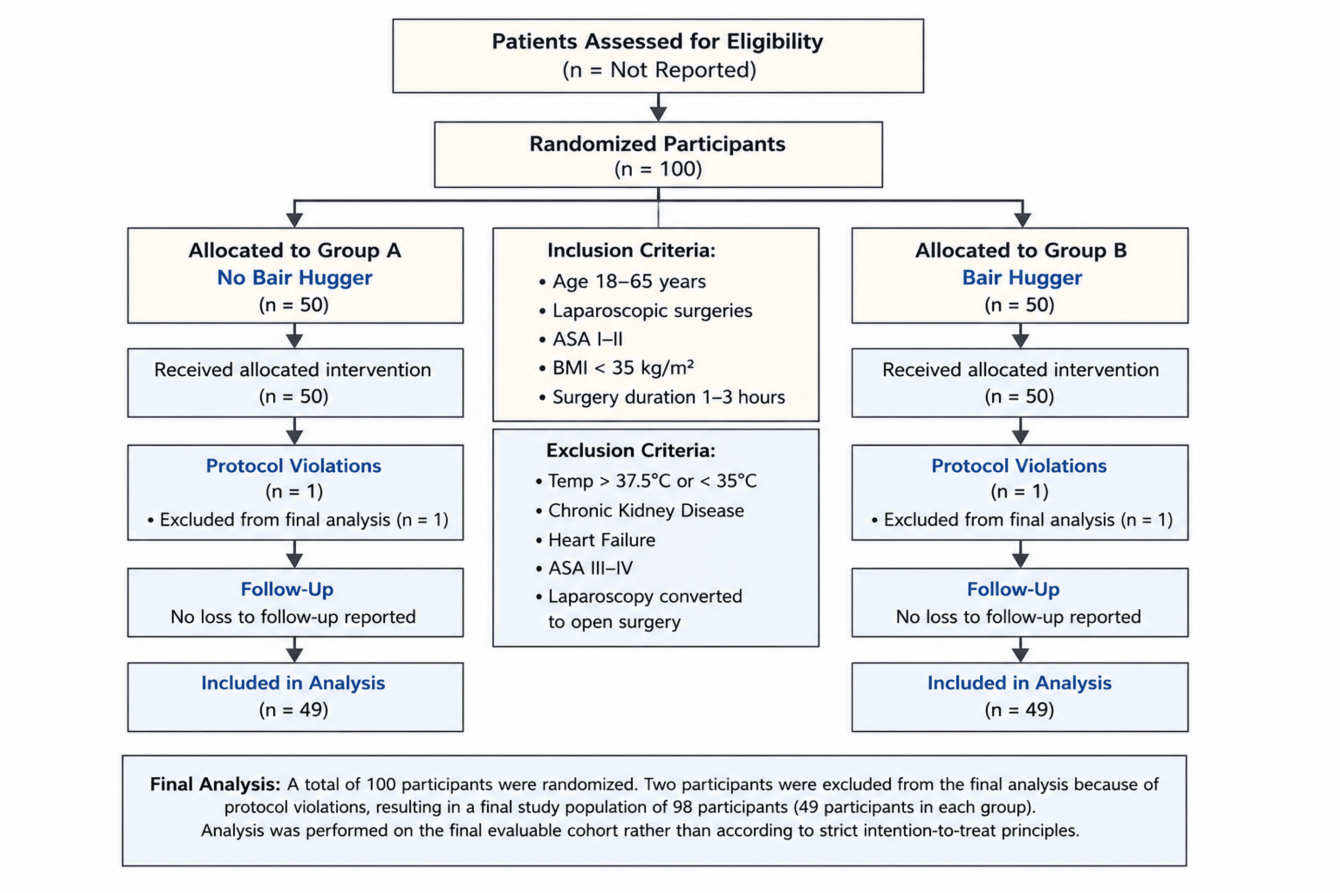
CONSORT diagram of inclusion and exclusion, with allocation into groups A and B

An independent statistician generated the random allocation sequence using a computerized random number generator. Allocation concealment was maintained using sequentially numbered, opaque, sealed envelopes. The envelopes were opened sequentially by the primary investigator only after completion of baseline participant assessment. Group A, the control group (No Bair Hugger), and Group B, the intervention group (with Bair Hugger), as shown in Figure 1. The participants were not informed of the group they had been allocated to. The FAW group received intraoperative warming with the Bair Hugger device, applied 5-10 minutes after induction and removed before extubation, while the control group did not receive active warming. The anesthesiologist managing the case was aware of allocation because of the visible nature of the intervention, while patients and postoperative assessors remained blinded.

Upon the patient's arrival in the OR waiting area, the body temperature was recorded initially with an infrared thermometer. All patients received standardized anaesthesia, and patients in both groups received approximately 500 mL of intravenous fluid warmed to 39 °C. The operating room temperature was maintained at 24 °C, and intravenous ondansetron (8 mg) was administered routinely at the end of surgery. Room-temperature carbon dioxide insufflation was used for all laparoscopic procedures, and no warmed CO₂ insufflation was employed; all patients, irrespective of group allocation, were covered with a K-Thermia warming blanket before induction. Premedication, induction with propofol, opioids, and atracurium, and maintenance with isoflurane in oxygen/air. Pneumoperitoneum was maintained at 12-14 mmHg. Core temperature was measured using a nasopharyngeal probe inserted immediately after induction, with recordings taken every 30 minutes intraoperatively until the end of the procedure, and again skin temperature was measured with an infrared thermometer upon arrival in the post-anesthesia care unit (PACU).

The outcomes measured were PONV and postoperative shivering. The PONV was measured by a 0-3 verbal descriptive scale ranging from 0 for none, 1 for nausea present, no vomiting, 2 for nausea present and vomiting once, and 3 for more than two episodes. The postoperative shivering was measured by a 4-point Bedside Shivering Assessment Scale (BSAS) from 0 to 3, where 0 = no shivering, 1 = mild shivering localized to the neck or thorax, 2 = moderate shivering involving the upper extremities, and 3 = severe generalized shivering involving the whole body. The endpoints were measured 30 minutes and 6 hours after arrival at the recovery room. Figure 1 shows the CONSORT diagram for this study.

Sample size and statistical analysis

The sample size was calculated based on the primary outcome of postoperative shivering. A previous randomized controlled trial evaluating FAW reported a postoperative shivering incidence of 20% in the control group and 0% in the FAW group [9]. Using these proportions, a two-sided significance level (α) of 0.05 and a statistical power of 90%, the required sample size was calculated using the World Health Organization (WHO) sample size calculator, which yielded 46 participants per group. To compensate for an anticipated 10% attrition rate due to potential dropouts or protocol deviations, the sample size was increased to 50 participants per group, resulting in a total sample size of 100 patients.

Data were analyzed with SPSS version 19 (IBM Corp, Armonk, NY, US). Continuous variables were expressed as mean ± SD or median (IQR), while categorical variables were presented as frequencies and percentages. The student’s t-test or Mann-Whitney U test was used for continuous data, and the chi-square or Fisher’s exact test for categorical data. A p-value <0.05 was considered significant. For the association between shivering and age, gender, type of surgery, and duration of anesthesia, analysis of variance (ANOVA) and Fisher tests were used.

## Results

A total of N=100 patients were recruited for the study. Two patients were excluded due to protocol violations (conversion from laparoscopic to open surgery), leaving N=98 patients for analysis (N=49 in the FAW group and N=49 in the control group). The analysis was performed on the final evaluable cohort rather than according to strict intention-to-treat principles. Patient flow is shown in the CONSORT diagram (Figure 1).

Baseline characteristics were largely comparable between the two groups (Table 1). Age, gender, BMI, ASA status, duration of anesthesia, and duration of surgery did not differ significantly. A small but statistically significant difference was noted in the mean pre-induction temperature, which was slightly higher in the control group (36.3 °C vs 36.1 °C, p = 0.001) (Table 2).

**Table 1:** Patient demographics

	Variable	Non-Bair Hugger study (N=49)	P-value
	Type of Surgery	-	0.252
	Laparoscopic cholecystectomy	37 (75.5%)	-
	Laparoscopic hernia repair	7 (14.3%)	-
Laparoscopic Hysterectomy	2 (4.1%)	0 (0%)	-
Laparoscopic Ovarian Cystectomy	1 (2.0%)	2 (4.1%)	-
Laparoscopic Nephrectomy	1 (2.0%)	0 (0%)	-
Laparoscopic Appendectomy	0 (0%)	3 (6.1%)	-
Age			0.179
18-20 years	1 (2.0%)	3 (6.1%)	-
21-30 years	5 (10.2%)	11 (22.4%)	-
31-40 years	10 (20.4%)	11 (22.4%)	-
41-50 years	11 (22.4%)	12 (24.5%)	-
51-65 years	22 (44.9%)	12 (24.5%)	-
BMI			0.873
18.5-24.9 (Healthy weight)	10 (20.4%)	10 (20.4%)	-
25-29.9 (Overweight)	22 (44.9%)	20 (40.8%)	-
30.0 and above (Obese)	15 (30.6%)	18 (36.7%)	-
Less than 18.5 (Underweight)	2 (4.1%)	1 (2.0%)	-
Gender			0.2
Female	29 (59.2%)	36 (73.5%)	-
Male	20 (40.8%)	13 (26.5%)	-
ASA status			0.658
I	13 (26.5%)	16 (32.7%)	-
II	36 (73.5%)	33 (67.3%)	-
Smoker			0.452
No	37 (75.5%)	41 (83.7%)	-
Yes	12 (24.5%)	8 (16.3%)	-

**Table 2:** Data on patients' temperature

Variable	Bair Hugger study (N=49)	Non-Bair Hugger study (N=49)	P-value
Temperature in the preoperative area (skin temp)			
Mean (SD)	36.1 (0.359)	36.3 (0.389)	0.001
Temp. after induction (30 mins) core			
Mean (SD)	36.0 (0.415)	36.1 (0.434)	0.287
Temp after induction (1 hour)			
Mean (SD)	36.1 (0.413)	36.0 (0.414)	0.191
Temp after induction (1.5 hours)			
Mean (SD)	36.2 (0.373)	36.0 (0.427)	0.013
Temp after induction (2 hours)			
Mean (SD)	36.3 (0.390)	36.1 (0.423)	0.123
Temp after induction (2.5 hours)			
Mean (SD)	36.3 (0.387)	36.2 (0.515)	0.622
Temp after induction (3 hours)			
Mean (SD)	36.2 (0.564)	36.2 (0.265)	0.763
Temp on arrival in PACU (skin temperature)			
Mean (SD)	36.2 (0.330)	36.0 (0.389)	0.006

The linear mixed-effects model demonstrated a significant effect of time (F = 8.48, P < 0.001), indicating that body temperature changed significantly throughout the perioperative period. The overall effect of the study group was not statistically significant (F = 3.36, P = 0.070). However, a significant Study Group × Time interaction (F = 17.77, P < 0.001) was observed. This indicates that temperature trajectories differed significantly between the Bair Hugger and Non-Bair Hugger groups over time. Patients receiving active warming demonstrated superior maintenance of perioperative normothermia (Table 3).

**Table 3:** Linear mixed-effects model results

Effect	F_value	P-value
Study Type	3.36	0.07
Time	8.48	<0.001
Study Type: Time	17.77	<0.001

The incidence of postoperative shivering was only 6.1% (N=3) in the FAW group at 30 minutes in the recovery room, while the incidence in the control group was significantly higher at 32.7% (N=16) (p-value = 0.004) at the same time interval. By six hours post-extubation, shivering was almost completely resolved in both groups, and the difference was no longer statistically significant (100% vs 95.9%, p = 0.475) (Table 4).

**Table 4:** PONV and shivering data PONV: postoperative nausea and vomiting

Variable	Bair Hugger study (N=49)	Non-Bair Hugger study (N=49)	P-value
Shivering Scale Assessment (At 30 mins in PACU)			
None (0)	46 (93.9%)	33 (67.3%)	0.004
Mild (1)	2 (4.1%)	9 (18.4%)	
Moderate (2)	1 (2.0%)	7 (14.3%)	
Shivering Scale Assessment (At 6 hours)			
None (0)	49 (100%)	47 (95.9%)	0.475
Mild (1)	0 (0%)	2 (4.1%)	
Moderate	0	0	
Nausea/Vomiting Scale (At 30 mins in PACU)			
Both nausea & vomiting are absent (0)	43 (87.8%)	37 (75.5%)	0.232
Nausea present, but no vomiting (1)	6 (12.2%)	11 (22.4%)	
Both nausea & vomiting are present (2)	0 (0%)	1 (2.0%)	
Nausea/vomiting scale (at 6 hours)			
Both nausea & vomiting are absent (0)	47 (95.9%)	45 (91.8%)	0.673
Nausea present, no vomiting (1)	2 (4.1%)	4 (8.2%)	
Both nausea & vomiting are present (2)	0	0	

Intraoperative core body temperatures were consistently higher in the FAW group compared to the control group. A statistically significant difference was noted at 1.5 hours after induction (36.2 vs 36.0 °C, p = 0.013) and upon arrival in the PACU (skin temperature 36.2 vs 36.0 °C, p = 0.006). At other intraoperative time points, temperatures were not significantly different. There was no association between shivering and the type of laparoscopic surgery, age, or gender. There was no statistically significant difference in the incidence of PONV between groups. Patients did not complain of vomiting in either group. Thirty minutes after extubation, nausea was observed in 17 (17.3%) patients, but only 4 of these patients shivered in the PACU. At six hours, only six patients complained of nausea, and all these patients belonged to the non-shivering group (Table 5).

**Table 5:** Shivering scale assessment (at 30 mins), and its predictors

	Mild (1) (N=11)	Moderate (2) (N=8)	None (0) (N=79)	P-value
Type of Surgery				
Laparoscopic Appendectomy	1 (9.1%)	1 (12.5%)	1 (1.3%)	0.177
Laparoscopic Cholecystectomy	7 (63.6%)	6 (75.0%)	60 (75.9%)	-
Laparoscopic Hernia Repair	3 (27.3%)	0 (0%)	13 (16.5%)	-
Laparoscopic Hysterectomy	0 (0%)	1 (12.5%)	1 (1.3%)	-
Laparoscopic Ovarian Cystectomy	0 (0%)	0 (0%)	3 (3.8%)	-
Laparoscopic Nephrectomy	0 (0%)	0 (0%)	1 (1.3%)	-
Age				
21-30 years	3 (27.3%)	2 (25.0%)	11 (13.9%)	0.217
31-40 years	2 (18.2%)	2 (25.0%)	17 (21.5%)	-
51-65 years	6 (54.5%)	1 (12.5%)	27 (34.2%)	-
18-20 years	0 (0%)	1 (12.5%)	3 (3.8%)	-
41-50 years	0 (0%)	2 (25.0%)	21 (26.6%)	-
Gender				
Female	7 (63.6%)	7 (87.5%)	51 (64.6%)	0.445
Male	4 (36.4%)	1 (12.5%)	28 (35.4%)	-
Duration of Anaesthesia				
121 mins-180 mins	2 (18.2%)	2 (25.0%)	28 (35.4%)	0.839
61 min-90 mins	2 (18.2%)	2 (25.0%)	15 (19.0%)	-
91 mins-120 mins	7 (63.6%)	4 (50.0%)	34 (43.0%)	-
More than 180 mins	0 (0%)	0 (0%)	2 (2.5%)	-

## Discussion

Shivering is one of the most distressing complications after surgery, and the incidence in laparoscopic surgeries is still about 40% despite the limited duration of anaesthesia [18]. Shivering is associated with discomfort, monitoring issues, increased oxygen consumption, increased CO2 production, and delayed discharge. Among other causes, intraoperative hypothermia is considered the strongest predictor of shivering. Several strategies have been used to prevent intraoperative hypothermia, which include FAW devices, heating blankets, warming intravenous fluids, maintaining higher room and PACU temperatures, and certain drugs.

Previous studies have shown that, in the absence of active warming measures, the greatest decline in intraoperative core temperature typically occurs within the first 30-60 minutes after induction of anesthesia [19]. Similarly, a decrease in temperature was observed in both groups at 30 minutes in our study. However, patients who subsequently developed postoperative shivering experienced a significantly greater reduction in core temperature at 60 and 90 minutes after induction compared with those who did not shiver (35.7 °C vs. 36.1 °C). Notably, core temperatures in the non-shivering group remained consistently above 36 °C throughout the intraoperative period, whereas temperatures in the shivering group fell below this threshold. Furthermore, patients who developed moderate shivering exhibited the lowest intraoperative temperatures compared with those who experienced mild or no shivering, suggesting a clear association between the degree of perioperative hypothermia and the severity of postoperative shivering.

Emran et al. used warm IV fluids for maxillofacial surgeries and were able to significantly reduce the shivering incidence from 42.9% to 11.7% [20]. Our study showed that the intraoperative forced-air warming helps maintain temperature and significantly reduces the incidence of postoperative shivering in PACU. Only 3 (6.1%) patients in the FAW group and 16 (32.7%) in the non-FAW group experienced shivering in the first 30 minutes in the PACU (p-value 0.004). A previous study also demonstrated the role of active warming in reducing redistribution and thermoregulatory stress [21]. The modest maintenance of temperature intraoperatively in the FAW group (≈0.2 °C) was sufficient to suppress shivering thresholds. Although the temperature difference was marginally higher in the FAW group in the operating room and recovery room, it does not completely explain the reduction in shivering rate. Incidence of shivering was higher in female patients (N=14) when compared with males (N=5), but it did not reach a significant level (p-value 0.445). A high rate of shivering was also noticed in patients whose surgery lasted more than 120 minutes.

Dedeoğlu et al. used active warming with a resistive carbon fibre underbody blanket in laparoscopic cholecystectomy patients and reduced the incidence of shivering in the active warming group (20.0% vs. 46.3%) [22]. Yin et al. looked at the effect of hypothermia and postoperative shivering in abdominal surgeries and found that the incidence of shivering and prolonged recovery time is higher in elderly patients, even with mild hypothermia [23]. The present study also demonstrates a higher incidence of shivering (N=7; 33.5%) in patients in the 51-65 age group.

Certain drugs are commonly used for the management of shivering in PACU. In one of the studies, preemptive doses of pethidine were also tried to prevent this complication, but were unable to demonstrate any benefit.

The effect of IOH on PONV has not been studied extensively, but there is limited evidence that IOH is associated with increased nausea incidence but has no effect on vomiting [8]. Although the incidence of nausea was slightly higher in the control group, no significant difference in PONV was observed between the groups, which is comparable to findings from other trials [24]. The multifactorial etiology of PONV, including anesthetic technique, opioid administration, and individual susceptibility, may explain why FAW does not consistently affect its incidence [25]. Reduced incidence of PONV may be because all our patients received antiemetics at the end of laparoscopic surgery.

The strengths of this study include its randomized design, standardized anesthesia protocol, and use of validated scoring systems. Limitations include its single-center setting, relatively small sample size, and short follow-up of only six hours. A small imbalance in baseline pre-induction temperature was also noted, which could have introduced bias, although the direction of the difference suggests that the true benefit of FAW may have been underestimated.

The present study also demonstrates the association of shivering with intraoperative hypothermia. Overall, the results support the use of forced air warming during laparoscopic surgery to improve early postoperative comfort by reducing shivering, even though it does not significantly influence PONV.

Limitations

The study was single-centered, and therefore, it might not be generalized to other types of patient populations. Most procedures lasted between one and two hours, with very few studies extending beyond. Therefore, the effectiveness and applicability of the Bair Hugger in prolonged surgery remain uncertain. Only patients were blinded, while the anesthesiologist collecting the data knew group allocation, which introduces observer bias. Postoperative shivering and PONV were assessed only up to six hours after surgery. Delayed PONV occurring between 6 and 24 hours may not have been captured and may provide additional information regarding delayed PONV. Baseline and PACU temperatures were assessed using an infrared thermometer because postoperative core temperature monitoring was not routinely available. This may have reduced the accuracy of postoperative temperature assessment. Prewarming has been shown to reduce redistribution hypothermia, but it was not part of the routine perioperative warming protocol at our institution during the study period.

## Conclusions

Intraoperative forced-air warming significantly reduces the incidence of postoperative shivering in patients undergoing laparoscopic surgery, but is unable to demonstrate a significant impact on postoperative nausea and vomiting. These findings support the use of forced-air warming devices as a safe and effective measure to prevent shivering, particularly in high-risk patients.
